# Indigenizing collaborative methods in studying human–water relations in the Syilx Okanagan Territory of British Columbia, Canada

**DOI:** 10.1177/14687941241306234

**Published:** 2025-01-15

**Authors:** Maria Correia, Sarah Alexis, Aleksandra Dulic

**Affiliations:** UBC Okanagan, Canada

**Keywords:** cross-cultural collaboration, Indigenous methodologies, co-design, capacity bridging, human–water relations, Syilx Nation

## Abstract

This article describes co-design and Indigenous methodologies applied in Waterways, Past, Present, and Future, a research inquiry and immersive media exhibition on human water relations carried out in the Syilx Okanagan First Nation territory of British Columbia, Canada. As a backdrop, we provide an overview of collaborative research, co-design, and Indigenous methodologies principles and then describe how these edicts were reflected in Waterway's praxis. The co-leadership of the research team, the cross-cultural interdisciplinary composition of the team, the decolonizing frame applied throughout the inquiry and design process, and the timeframe for team members to carry out personalized and collective multi-layered preparation were key ingredients in the application of Indigenous methodologies. Co-design principles observed in Waterways included prioritizing design justice and incorporating reflexivity and flexibility, iteration, and emergence in the design process. These methodological considerations can lead to more impactful co-design and cross-cultural research collaboration in Indigenous settings, which is currently a priority in Canada's ongoing reconciliation process.

## Introduction

Many of today's complex societal problems transcend the capacity of any single entity to revolve, thereby demanding collaboration across organizational, sectoral, and jurisdictional boundaries. The COVID-19 pandemic and the extraordinary efforts to develop a vaccine illustrate how cross-sector collaboration can achieve successful and consequential outcomes. Climate change, poverty, and migration are other examples of global issues that require collective actions. The complexity and wicked nature of these issues and their physical, political, economic, and cultural interdependencies mean that multiple actors must pool competencies and resources to find and implement solutions (Ansell and Gash, 2008; IPCC, 2021; Wood and Gray, 1991).

The academic literature on collaboration is broad and multidisciplinary, drawing from fields such as organizational psychology, sociology, management, and communication, among others (Cummings and Kiesler, 2005; Huxham, 2003; Inkpen and Tsang, 2005; Sawyer, 2017). Collaboration in research, including the use of participatory design and related practices, has gained legitimacy and is increasingly employed as a strategy of inquiry in the academic sphere (Bjögvinsson et al., 2012; Cornwall and Jewkes, 1995; Cummings and Kiesler, 2005). The need for interdisciplinary approaches to collaboration is also widely acknowledged in academia, albeit often challenging to implement in practice (Klein, 2010; Robinson, 2008; Stokols et al., 2008).

In 2017, a group of Indigenous and non-Indigenous scholars and community partners came together to explore the fragile relationship between people and water in the traditional territory of the Syilx Okanagan First Nation, located in British Columbia (BC), Canada. The 4-year collaborative research project, titled *Waterways, the Past, Present and Future*, aimed to promote sustainable water practices in one of the most water-stressed regions of Canada through an interactive, immersive museum exhibition that exposed the public to diverse values, worldviews, and perspectives on water. Ultimately, waterways sought to foster cross-cultural engagement and create a space for identifying common ground on water stewardship across cultures.

In this paper, we examine the collaborative methodologies applied in waterways, with the goal of understanding how to facilitate cross-cultural research collaboration in a real-world Indigenous context. The paper comprises two main parts. The first provides an overview of Western-oriented collaborative research methods, including co-design and design justice alongside Indigenous research methodologies. The second section examines how these methods and methodological principles were manifested in the waterways research and museum installation. We follow with the discussion and conclusions sections.

Throughout the paper, the term Okanagan refers to the name of the Syilx Okanagan people and to the geographic location (valley, lake, and river) of Syilx traditional lands. The word Indigenous is defined here as the “profoundly undisruptive association with one place that was developed over many millennia by a people who shared that place as *member* of its flora and fauna” (Armstrong, 2010: 85‒86). Indigenizing in this context is defined as “the process of bringing Indigenous worldviews, values, and practices into the design, conduct, and dissemination of research” and involves a commitment to ethical relationships with Indigenous communities and recognition of the ongoing impacts of colonization (Kovach, 2010: 22). In Canada, the word “Indigenous” encompasses First Nations, Inuit, and Métis peoples (Indigenous Foundations, 2009). The term “First Nation” refers to Aboriginal peoples of Canada who are ethnically neither Métis nor Inuit, whereas “Aboriginal” is a term applied when referring to the first inhabitants of Canada, including First Nations, Inuit, and Métis peoples. These terms are capitalized in accordance with guidelines for writing about Indigenous Peoples (Younging and Ebscohost, 2018).

## Western methodologies

### Collaborative research

The scholarly literature on collaboration is vast and reaches into several fields. In management and administration, early writing on collaboration focused on dynamic processes in complex organizational and institutional settings (Holley et al., 2012). One of the most cited scholars on the topic, Barbara Gray, defines collaboration as the pooling of tangible and intangible resources by multiple individuals or groups to solve a set of problems that neither can solve on an individual basis (Gray, 1985). Gray sets out a process model for collaboration that includes three phases—problem-setting, direction-setting, and structuring—and proposes conditions for achieving collaboration based on organization theory, policy analysis, and organizational development. In Gray's (1989) comprehensive overview of collaboration in the context of multiparty problems, she notes the following considerations: the complexity of groups and individuals involved and the manner in which these entities change and their proximity; the interdependence of these groups and individuals and the power dynamics between them; the perceived legitimacy of collaboration and the characteristics of the conveners; and historical power relations, including trust.

In the environmental realm, collaboration and multi-actor collaboration is seen as imperative for building social-ecological resilience and adaptive capacity (Armitage et al., 2007; Berkes, 2009; Folke et al., 2005), as well as addressing the inherent political, jurisdictional, and cross-cultural characteristics of social-ecological systems (Bodin, 2017). Diverse groups and individuals can bring unique knowledge and understandings gained through experience with different aspects of the system at distinct spatial and temporal scales (Berkes, 2004; Manfredo et al., 2014; Prell et al., 2009; Reed, 2008). Cooperative, collaborative, and collective action is seen as the best approach to deal with common-pool resources (Ostrom, 1990) such as forests, watersheds, fisheries, and protected areas, which often include Indigenous groups (Castro and Nielsen, 2001).

Collaboration in research has been defined as an inquiry that involves participants in a situation under examination, with mutual learning taking place between researchers and participants (Müller et al., 2012). Collaborative research is a powerful and deep tradition of inquiry, with theorists and practitioners such as John Dewey, Paolo Freire, Raymond Williams, and Donna Haraway providing important philosophical underpinnings (Zamenopoulos and Alexiou, 2018). Haraway (2020) explores the concept of “situated knowledges,” which highlights the social, political, and historical context in which knowledge is produced. She argues that collaborative and interdisciplinary research is essential for developing more nuanced and accurate understandings of complex issues. Theorists and practitioners John Dewey, Paolo Freire, and Raymond Williams contributed to this research tradition by emphasizing collaboration in education and cultural analysis; they recognized that learning and culture were social processes that required the active participation of multiple actors, and advocated for a more democratic and inclusive approach to research and education (Dewey, 1916; Freire, 2020; Williams, 1980).

Collaboration in academia across disciplines and boundaries has become widely accepted and increasingly commonplace given the complexity of today's societal problems, which demand innovative solutions that pool knowledge across scientific disciplines (Robinson, 2008; Stokols et al., 2008; van Rijnsoever and Hessels, 2011). Inter-, multi-, and transdisciplinarity offers the potential to combine knowledge from reductionist disciplines and gain a more holistic understanding of complex issues. From a practical standpoint, working across disciplines can help identify workable solutions based on recognizable behavioral patterns while also considering the intricate nature of the problem at hand (Newell, 2003). Challenges to conducting research across disciplines persist, however, for several reasons. These include communication and language barriers (Hackett et al., 2008; Klein, 2008); different assumptions, values, and goals, which can make it challenging to find common ground (Bergmann et al., 2012; Klein, 2008); institutional barriers, including access to funding (Bergmann et al., 2012; Robinson, 2008; Stokols et al., 2008); and distinct theoretical assumptions and methodological orientations (Huutoniemi et al., 2010; Stokols, 2006). Biophysical sciences, for example, gravitate to quantitative research compared to social sciences, which apply both quantitative and qualitative and interpretive validation approaches (Brown et al., 2015; Pohl, 2005).

The nuanced distinctions between interdisciplinarity, multidisciplinarity, and transdisciplinarity are pertinent when engaging across Western science and Indigenous knowledge systems, which are inherently boundary-crossing (Moewaka Barnes et al., 2021; Wilson, 2008). Interdisciplinarity involves the fusion of knowledge and methodologies from diverse disciplines to address shared issues, enabling the synthesis of approaches that contribute to a more comprehensive understanding of the issue in question (Huutoniemi et al., 2010; Klein, 2010). A biologist, for example, could be collaborating with an Indigenous community to comprehend local ecological knowledge. Multidisciplinarity, on the other hand, involves juxtaposing different disciplinary perspectives side by side to address specific issues without necessarily integrating them (Klein, 2010). In an Indigenous context, this may encompass a comparative examination of Western and Indigenous knowledge systems, acknowledging their distinct yet complementary nature (Houde, 2007; Turner et al., 2000). Transdisciplinarity takes collaboration a step further. It aims to transcend individual disciplines entirely and promote knowledge co-creation relevant to both scientific and societal challenges (Klein, 2008). These include wicked problems that are characterized by high levels of complexity, uncertainty, and value conflicts (Harris et al., 2010). It entails integrating various types of knowledge from different disciplines and stakeholders and applying this knowledge collaboratively to real-world issues (Bergmann et al., 2012; Jahn et al., 2012). In the context of Western science and Indigenous knowledge systems, transdisciplinarity often involves partnerships among Western scientists, Indigenous elders, community members, and policymakers (Christie, 2006; Harris et al., 2010; Klein, 2004). Establishing external alliances through transdisciplinarity builds understanding through multiple ways of knowing and doing and embraces complexity through connection (Robinson, 2008).

### Participatory research

Participatory research is a collaborative approach commonly used by scholars and practitioners across the behavioral and social sciences (Kindon et al., 2007; McIntyre, 2007; Schubotz, 2020). Participation is considered an orientation to inquiry and, thus, a methodology (Bergold and Thomas, 2012; Bradbury-Huang, 2015). Broadly defined, participatory research is a practice undertaken *with* rather than *on* people (Schubotz, 2020). Agency, representation, and power lie at the core of the participatory inquiry (Cornwall and Jewkes, 1995). Participatory research requires a high degree of self-reflexivity and reflection on the situated engagement, given that participants bring their views and perspectives into knowledge generation. To reach a consensus or mutual agreement, all participants, including the researchers, must, to some extent, disclose and create common ground for their epistemological foundations, thus requiring researchers to acknowledge their role as the instrument of the inquiry (Bergold and Thomas, 2012).

Participatory research covers a range of approaches, with methods often drawn from conventional and mainstream practices that involve different levels of engagement (Cornwall and Jewkes, 1995). Vaughn and Jacquez (2020) review 27 frameworks, orientations, and approaches to participatory research and find that these share the common goals of achieving real-world impact and generating knowledge. They also identify five “choice points” in research where decisions on the degree of participation are made. These, in turn, guide the selection of methods and tools to use in the inquiry. The choices are (1) inform (information is provided to the community), (2) consult (input is obtained from the community), (3) involve (researchers work directly with the community), (4) collaborate (community is a partner in research) and (5) empower (community leads research in decision-making). Arnstein's (1969) ladder of participation, a common reference point both in research and in practice, identifies eight rungs on a metaphorical ladder, which range from the lowest level (passive dissemination of information) to the highest level (citizen control of the process).

Several challenges are associated with participatory research. Power dynamics can be an issue, for example, as community members may feel intimidated or less knowledgeable than researchers, which can affect the quality of their participation (Hacker, 2013; Wallerstein et al., 2019). Participants also need a safe space to speak freely and comfortably to express their opinions and views, which can be a major impediment in communities that have faced historical marginalization, including Indigenous communities (Bergold and Thomas, 2012; Cargo and Mercer, 2008). Delineating the community that is meant to participate is a further challenge: who is the community, who should be included, and who should not, which individuals or groups should or must participate (Bergold and Thomas, 2012; Hacker, 2013). In contrast to conventional research, where the interaction is neutral, the nature of the relationship between the researcher and the community or individuals partaking in the inquiry has come into question in the participatory research approach (Bergold and Thomas, 2012; Wallerstein and Duran, 2008).

### Co-design

Co-design, also known as participatory design, is a form of collaborative research that involves active collaboration between researchers and participants throughout the research process. Zamenopoulos and Alexiou (2018) define co-design as a process in which individuals come together to conceptually develop and create something that responds to issues of concern, despite or because of their distinct needs, skills, and knowledge. A basic premise of co-design is designing *with* the people, based on their interests, instead of designing *for* the people (Burkett, 2012). Co-design is related to the concept of co-creation, in that collective creativity is applied across the design spectrum (Sanders and Simons, 2009). Co-design has broad applications, having been used both in designing material goods and products and in improving services, including social services (Sanders et al., 2018).

In academic inquiry, co-design may include researchers, practitioners, and community members who are involved throughout the design process, from problem definition to the production process to the finished artifact or solution (Hedditch and Vyas, 2023). Co-design research principles include *equity and respect*, to account for any power imbalances and differences in values, knowledge, and expertise; *flexibility* to accommodate the perspectives and priorities of participants; *iteration*, to ensure continuous feedback, learning, adjustment, and relevance; and *empowerment*, to create a sense of ownership of the design and the possibility of meaningful change (Cantu and Selloni, 2013; Lilley, 2009; Sanders and Stappers, 2008, 2014). Communication, synchronicity, coordination, reflectivity, knowledge creation and integration, and shared understanding of content and process are all important features of co-design (Tessier and Mithra, 2019), as are risk-taking and consensus-building given its collective nature (Kvan, 2000). Design solutions may include tangible products, systems, and infrastructures or intangibles such as processes, strategies, and policies (Zamenopoulos and Alexiou, 2018).

### Co-design as reflective practice

Co-design is rooted in the practice of reflection-in-action (Schön, 2017), where the “reflective practitioner” inquiries into the real-world design situation and simultaneously integrates reflection, action, and implementation. This concept builds from a pragmatist orientation to common sense, practical knowing, and the facts of experience (Dewey, 1903; James, 1907; Peirce, 1991). The reflective practitioner conducts an artful inquiry into situations of uncertainty, which enables the discovery of contextual knowledge critical for culturally specific products and technologies (Dieleman, 2012). The “contextual rationality” of the reflective practitioner enables representations of the community within design solutions (Schön, 2017).

Co-design is understood to be interactive and experiential (Wakkary, 2009) and therefore encompasses the complex relationship between designers, the known and lived world, and design actions. This concept, also known as experimentation-in-action, shapes the processes and goals through framing and reframing a problem as a way of working towards the best design solution (Gedenryd, 1998; Schon, 1984). Experience is understood as the actions humans do in the world that hold value and are conceptually meaningful (Alexander and Dewey, 1987). The influential American philosopher, psychologist, and educational reformer Dewey (1934) situates learning through concrete lived experience within the context of being involved in a temporal, open-ended physical world. Experiences, situations, and nature are understood as continuous and qualitative, beginning and ending in a larger world of immediate experience rather than only in a reflective space. Furthermore, aesthetic experiences allow possible meanings and values to emerge in situations that are relational and contextual. Acquiring empirical knowledge through engagement and experience reveals contextual and situated meaning, thus bringing together ways of knowing through experience that unite heart and mind, affect and cognition, art, and science, rather than keeping them in opposition (Dewey, 1934).

### Design justice

A general critique of conventional design is that it privileges dominant cultures. Costanza-Chock (2020) argues for “design justice,” which fundamentally implies that marginalized communities lead the process for which the design is intended. Design justice explores the relationship between design, power, and social justice. According to Costanza-Chock, design justice is a practice and philosophy in which all participants are considered to be experts with unique and invaluable contributions. It recognizes that there are multiple ways of bringing knowledge and skills to the design process, including technical and professional skills and lived experience. Designers, in turn, play the role of facilitators rather than experts in framing solutions and bringing technical and evidence-based knowledge for ongoing learning and adaptation. Other key elements of this practice include using design to heal and empower communities and seek liberation from exploitative and oppressive systems, bringing diverse ways of knowing to create sustainable community-centered nonexploitative and ecologically-grounded solutions, bringing to the design process the voices of those who are most impacted by the design, honoring traditional, Indigenous and local knowledge and practices, prioritizing design impact on the community over the intention of the designer, and focusing on both process and product (Costanza-Chock, 2020). Since design is present in virtually every industry and affects societal structures, design justice concepts help us to understand the interplay between design, power, and social outcomes and reflect on how to avoid reinforcing and reproducing societal and structural inequalities.

## Indigenous methodologies

The word itself “research” is probably one of the dirtiest words in the indigenous world's vocabulary. When mentioned in many indigenous contexts, it stirs up silence, it conjures up bad memories, it raises a smile that is knowing and distrustful. (Linda Tuhiwai Smith, 2012)

Smith's (2012) influential publication on decolonizing methodologies provides a cautionary note and a reminder of the complexity and sensitivities of conducting research in an Indigenous context. Indigenous scholars researching and writing on research methodologies provide invaluable guidance on how engagement with Indigenous communities should take place. They point to important principles such as taking an Indigenous “paradigmatic perspective,” acknowledging Indigenous knowledge systems, adopting a decolonizing lens in the inquiry, considering who should conduct the research and/or engage with the Indigenous community, and ensuring researchers understand the cultural protocols, values, and beliefs of the respective Indigenous group. This section elaborates on these points based on the writings of these scholars.

### A paradigmatic approach

One of the resounding themes from scholarly writings on methodologies for engaging the Indigenous community is understanding the (research) paradigm. A research paradigm is defined by Wilson (2001: 175) as “a set of beliefs about the world and about gaining knowledge that goes together to guide your actions as to how you’re going to go about doing your research,” the four main elements of which are: ontology, a belief in the nature of reality, “your way of being, what you believe is real in the world”; epistemology, “how you think about that reality”; methodology, “how you are going to use your ways of thinking (your epistemology) to gain more knowledge about your reality”; and axiology, “a set of morals or set of ethics.” Kovach (2010) notes that applying a paradigmatic approach means that the research flows from an Indigenous belief system and has at its core a relational understanding and accountability to the world. Comparing Indigenous paradigms to Western ones, Wilson (2001) explains that dominant Western paradigms are built on the premise that the researcher is an individual searching for knowledge: knowledge is something that is gained, and thus, knowledge cannot be owned by an individual. This way of thinking contrasts with an Indigenous paradigm and the fundamental belief that knowledge is relational and shared with all creation and community: “it is with the cosmos, it is with the animals, with the plants, with the earth” (Wilson, 2001: 176, 177). Indigenous paradigms reflect Indigenous epistemologies, which hold a non-human-centric relational philosophy (Kovach, 2010).

### Indigenous knowledge(s)

Understanding the nature of Indigenous knowledge, which is unique to given cultures, localities, and societies and is acquired by Indigenous peoples through daily experience, is an important element of Indigenous methodologies and engagement (Steinhauer, 2002). Indigenous knowledge is defined as “the people's cognitive and wise legacy as a result of their interaction with nature in a common territory” (1999: 62), as local, holistic, and oral (Castellano, 2000) and as personal, oral, experiential, and holistic (Hart, 2010). “Ways of Knowing” are part of Indigenous knowledge systems, beyond the facts and information taught and learned (Martin and Mirraboopa, 2003). Kovach (2010) refers to Indigenous knowledge as plural, that is, knowledge(s), and asserts that it is a specific “way of knowing” based on oral tradition for sharing knowledge. She also maintains that story is central to Indigenous knowledge and methodology, noting the “inseparable relationship between story and knowing” (94). “The interrelationship between story and knowing cannot be traced back to any specific starting time within tribal societies, for they have been tightly bound since time immemorial as a legitimate form for understanding” (95). Steinhauer (2002) states that Indigenous knowledge comes from multiple sources, including traditional teachings, family knowledge, empirical observation, and revealed knowledge through dreams, visions, cellular memory, and intuition.

### Decolonizing research

Indigenous research often includes or should include a decolonizing frame (Chilisa, 2017; Coombes et al., 2014; Kovach, 2010, 2021; Lavallée, 2009; Wilson, 2001). Smith's pivotal book on the subject, originally published in 1999, challenges traditional Western approaches to research, calling for the “decolonization” of methodologies and a new agenda of Indigenous research (Smith, 2012). Decolonization in this context is about critically analyzing and questioning the underlying assumptions, motivations, and values that inform research practices (Smith, 2012). Chilisa (2017), in writing on the African Indigenous context, argues that methodologies rooted in African philosophies, worldviews, and history, bring alternative ways of conducting research to the academic discourse. Such methodologies question academic and methodological imperialism and highlight problem and solution-driven research agendas. Kovach (2021) notes that decolonizing perspectives and Indigenous epistemologies emerge from different paradigms. She explains that an Indigenous paradigm is centered on Indigenous knowledge(s), whereas the decolonization of methodologies is a critical theory found within the transformative paradigm of Western tradition. An Indigenous paradigm, Kovach asserts, should have a decolonizing perspective.

Thambinathan and Kinsella (2021) propose four elements to guide researchers in employing a decolonizing lens: first, practicing creative reflexivity by questioning epistemological assumptions, the positionality of the researcher, and power dynamics, including any hierarchical barriers between the researcher and research participants; second, applying reciprocity and respect for Indigenous self-determination, which in addition to the broader aim of the research implies requesting consent at different stages of the research and listening affectively and attentively to Indigenous research participants; third, seeking to understand Indigenous “ways of knowing” by endeavoring to unlearn and re-imagine how we construct, produce and value knowledge; and fourth, embracing a transformative praxis, through the research objectives broadly and by creating a space for discussing decolonizing practices throughout the inquiry.

### Indigenous methods

Indigenous research and data collection methods are as diverse as the communities using them and can encompass storytelling, research circles, conversations, and journaling (Kovach, 2021). There is a continuum of ways to access information, from internal knowledge(s) that guide the research to external knowledge(s) from others (Kovach, 2010). According to a systemic review conducted by Drawson et al. (2017), Indigenous research methods shared the following characteristics: (1) contextual reflection, that is, researchers situate themselves and the Indigenous individuals with whom they were collaborating in the research process; (2) Indigenous participants are included in the research process in a way that is respectful and reciprocal as well as decolonizing and self-deterministic; (3) Indigenous “ways of knowing” are prioritized; (4) Indigenous communities determine the direction of the research and approaches used; and (5) values of the Indigenous group involved are reflected throughout the research method.

Some Indigenous researchers have employed mixed Indigenous-Western methodology as their approach. Kovach (2010), for example, combined a conversational method congruent with an Indigenous paradigm with the Western-grounded theory method to organize her research data. Lavalliée (2009) used two qualitative research methods in her inquiry: sharing circles to capture people's experiences and an Indigenous art-based research method influenced by the Western Photovoice approach. The integration of Indigenous and Western methodologies, however, has been a topic of debate and critical reflection among many Indigenous researchers. These debates relate to the importance of respecting and understanding the epistemological and ontological foundations of Indigenous knowledges and applying superficial or inappropriate blending with Western paradigms (Battiste, 2017; Kovach, 2009; Smith, 2012; Wilson, 2008).

Indigenous research methodology obliges researchers to carry out personalized multi-layered preparatory work, including cultural self-identification and clarification of the purpose and motivation of the research inquiry (Kovach, 2009). Different from Western methodologies, Indigenous research frameworks ask researchers to be clear about both the academic and personal purpose behind their studies, including asking about the purpose of the research, the researcher's motivation, and the contribution of the research back to the community (Drawson et al., 2017; Kovach, 2009). The self-introduction protocol is a part of this process, as Kovach explains: “It shows respect to ancestors and allows community to locate us” (2009: 110). Situating self or self-location anchors knowledge within our own personal experiences, and these, in turn, influence our interpretations as researchers. It also builds trust, reciprocity, and rapport between the researcher and those being researched (Kovach, 2009).

The relational element of Indigenous-led research is central to methodology, a concept referred to as “self-in-relation” (Graveline, 2000) and “researcher in relation” (Kovach, 2021). As noted by Kovach (2021: 111): “Tribal epistemologies cannot be disassociated with the subjective. Tribal epistemologies are a way of knowing that does not debate the subjectivity factor in knowledge production – subjectivity is a given. To embrace Indigenous methodologies is to accept subjective knowledge.” Cultural grounding, defined as “the way culture nourishes the researcher's spirit during the inquiry and how it nourishes the research itself,” is another element of situating self with the research (Kovach, 2021: 116). Battiste (2005) notes that Indigenous knowledge is based on subjective experiences and is grounded in the relationships between people, the environment, and the spiritual world, it includes time-tested approaches that foster sustainability and environmental integrity.

### Indigeneity and the researcher

Indigenous scholars pose important questions about who engages and conducts the research. For example:

Whose research is it? Who owns it? Whose interests does it serve? Who will benefit from it? Who has designed its questions and framed its scope? Who will carry it out? Who will write it up? How will its results be disseminated? (Smith, 2012: 5)

These queries are relevant, especially considering Indigenous methodologies are relational. As Steinhauer (2002: 72, 73) puts it: “How could anyone who is not Indigenous have the Indigenous knowledge that is required?” Weber-Pillwax (1999) also questions the indigeneity of the researcher, given political, social, and personal implications. Smith (2012) argues for a research agenda within Indigenous communities only, contending that the role of the non-Indigenous researcher is marginal for decolonizing methodologies. According to Smith, research carried out through “imperial eyes” has historically oppressed Indigenous peoples and suppressed Indigenous knowledge (Smith, 2012). Independent of who the researcher is, Weber-Pillwax (1999) suggests that Indigenous methodologies consider the following principles:

- the *interconnectedness* of all living things;
- the *motives and intentions of the research* and how these aspects impact the community;
- the foundation of Indigenous research, which must lie within *the reality of lived Indigenous experience*;
- the theories developed or grounded, which must be supported by *Indigenous epistemology as it is lived out and formed in the community*;
- the *transformative nature of research*, which occurs as a natural result of personal internalizations of learning;
- the sacredness and responsibility of maintaining *personal and community integrity;* and
- the *recognition of languages and cultures as living processes*, with Indigenous researchers having the responsibility to maintain and renew connections with ancestors and Indigenous people through the practice of these ways.

Steinhauer (2002: 73) asserts that to adhere to Indigenous methodological principles, a researcher must know the cultural protocols, values, and beliefs of the Indigenous group associated with the study; a researcher must ensure that “the three R's – respect, reciprocity, and relationality – are guiding the research.”

Having discussed key concepts related to collaborative research, co-design, and Indigenous methodologies, the following section introduces Waterways and discusses how these methodological approaches were reflected in this real-life project.

## Waterways: past, present, and future

### Waterways context

One of the strongest points raised by the majority of Syilx Okanagan community members is that we need to act now and be true leaders in water protection and management.—Syilx siwɬk^w^ (water) Strategy. (ONA, 2021: 8)

**Figure 1.**
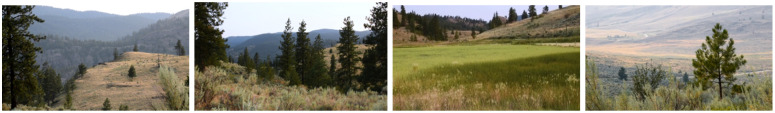
Images of Okanagan landscapes.

From 2017 to 2021, a team of Syilx and non-Indigenous academics and community partners collaborated in an inquiry and museum exhibition on human-water relations in the Okanagan Valley of BC, Canada, entitled Waterways, Past, Present, and Future. Water is a persistent concern in the Okanagan. The region, which is classified as semi-arid (see Figure 1), has the lowest per-person water availability in Canada, yet domestic per capita water use is more than twice the national average (OBWB, 2011). A comprehensive water supply and demand study carried out by Okanagan Basin Water Board confirms that drought and water shortages are a significant threat to this region, and climate change scientists are predicting that, given current water use patterns and projected population growth, the valley will be facing significant and persistent water shortages by mid-century (Cohen et al., 2006).

Waterways was carried out on the territory of the Syilx (Okanagan) First Nation. The Syilx peoples, a trans-boundary tribe separated at the 49th parallel by the border between Canada and the US, have resided in the Okanagan region for millennia. The Syilx peoples have an intrinsic relationship with siwɬk^w^ (water). They equate water with life and consider water their sacred relation, which they have an obligation to protect and keep healthy to ensure resiliency and relationship to the tmix^w^ (land) (ONA, 2021). The Syilx Water Strategy (2021) recognizes the need to promote unity and collaboration with non-Indigenous neighbors and partners in Syilx Okanagan Territory to uphold their inherent responsibilities to siwɬk^w^ (water).

### The Waterways research and exhibition

“Waterways Past, Present, and Future” is a 4-year undertaking involving the University of British Columbia Okanagan (UBCO), Syilx Knowledge Keepers and local partners. The research and exhibition, collectively referred to as Waterways, are aimed at fostering sustainable water practices by exploring the nature of human-water relationships in BC's Okanagan Valley. While the exhibition's primary audience is the general public, its focus is on engaging school-age children and the Syilx community, given its Indigenous content. The Waterways exhibition opened in September 2021 at the Okanagan Heritage Museum in Kelowna, BC and is currently touring in the Okanagan Valley.

The Waterways exhibition features Syilx-led best practices in water management and social-ecological resilience, including the return of sockeye salmon to the Okanagan waterways and riparian restoration along Shingle Creek and Okanagan River. To communicate these stories in an immersive museum installation, the research team interviewed Syilx Knowledge Keepers, community members, and Western-trained experts, as well as recorded audio and video documentation of the environmental and cultural detail of the Okanagan landscape, soundscape, and community. During the interviews, participants discussed the significance of water, good practices in water ecosystems management and sustainability, new co-management arrangements for caring for our water and ecosystems, and lessons from these practices for the future. In total, 30 interviews and four focus groups with 32 participants were conducted, including 11 interviews carried out by a Syilx scholar with Syilx members of the Colville Confederated Tribe from Washington State in the USA. Interviews were semi-structured and open-ended to encourage conversation, as well as digitally recorded. In accordance with Syilx practice and protocol, each engagement involved a small gift to acknowledge the relationship and show gratitude and respect for the insights being offered.

### The research team

The research team comprised a large multidisciplinary group of artists and scientists, both Indigenous and non-Indigenous, spanning the fields of Indigenous studies, fine arts, media studies, social and environmental anthropology, environmental science, computer science, complexity sciences, and architecture, among others. The team's leaders brought deep experience in co-design, immersive technologies, participatory experiential design, complexity science, and Indigenous research methodologies. A Syilx Knowledge Keeper and eminent Canadian scholar co-led the project, which brought legitimacy and expertise to the research and ensured that Syilx traditional ecological knowledge (TEK) would be a foundational knowledge domain. Other Syilx scholars worked with non-Indigenous scholars to provide guidance and oversight at key junctures, including on methods and exhibition design, and conducted the interviews with Syilx Knowledge Keepers in Washington state.

A mixed two-person Syilx*-*non-Indigenous team carried out and analyzed Waterways interviews. Having a Syilx scholar as part of the interviewing duo opened doors to the Syilx community, helped build a trusting research environment and comfortable interview setting, provided a sense of openness, rapport, and confidentiality, and helped gain cultural understanding and nuance from interview accounts. The mixed team protocol brought a bi-cultural theoretical perspective to interpreting and meaning-making of interview data. The blending of Syilx and Western worldviews in the analytical process, at times, led to significantly different interpretations of interview excerpts. This divergence, while challenging at times, proved beneficial overall as it facilitated valuable cross-cultural learning and deepened our comprehension. The goal was not to come to a consensus on the interpretations but to honor and showcase the unique ways of knowing and understanding of the data from both worldviews. This difference in perspectives sparked growth among the researchers and enriched the project's intellectual fabric, highlighting the positive impacts of embracing diverse worldviews in research.

The museum installation reflects the contributions of the cross-cultural team, integrating architecture, graphic design, immersive design, multimedia, and technology to share multilayered narratives representing the exhibition's core concepts of human-water relations. The importance of bridging intercultural and transdisciplinary ways of knowing is reflected in recorded audio and video of interviewees and documentation of the environmental and cultural detail of the Okanagan landscape, soundscape, and community.

### Waterways exhibition spaces—a fusion of Western and Indigenous perspectives

**Figure 2.**
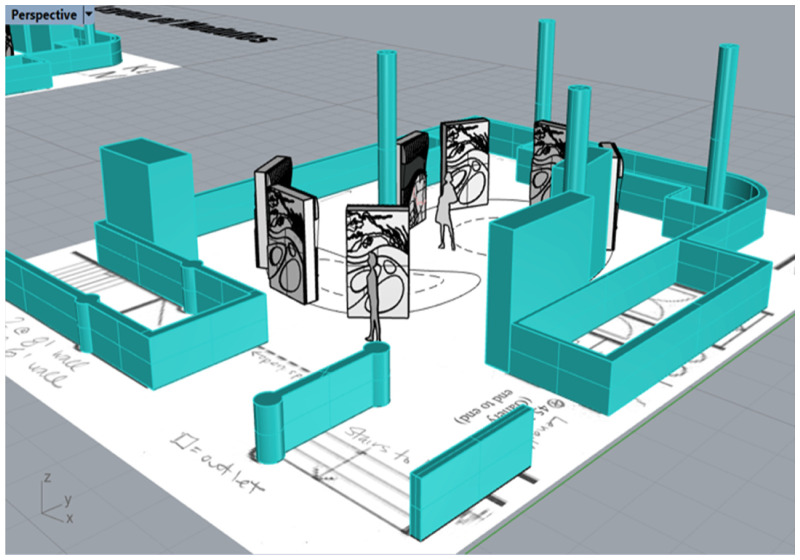
Drawing of Waterways exhibition floorplan. Drawing by Sepideh Saffari.

**Figure 3.**
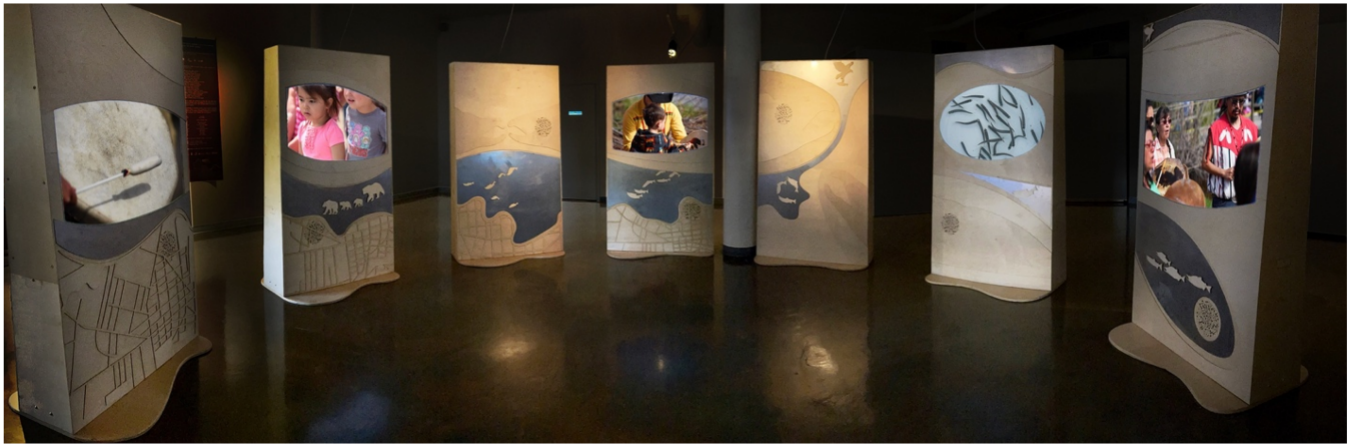
Waterways exhibition inner space. Photo by Sepideh Saffari.

Spatially, the museum installation includes a circular inner space comprising five video screens that feature continuous accounts from Syilx Knowledge Keepers and Western experts discussing the meaning of water and water stewardship (Figure 2). The narratives are superimposed with audio and video of Okanagan water, land, and soundscapes (Figure 3). The space was designed using a multi-channel sound and video media system, which was aimed at immersing, provoking, destabilizing, transforming, and moving museum visitors to reflect and take responsibility for water (Thorogood et al., 2022).

The outer area of the exhibition includes computerized interactive screens of 3D virtual worlds of the Kelowna area surrounded by displays of Indigenous Syilx teachings and wisdom on human-water relations, resilience, and water values. The 3D virtual worlds show the ecological changes to flood plains, wetlands, and riparian habitats around Mill and Mission Creek since colonization. Screens are interactive and depict the diversity of life in the Okanagan during this era, including plant, animal, and insect species indigenous to the region, which visitors can experience through touch-screen interfaces.

**Figure 4.**
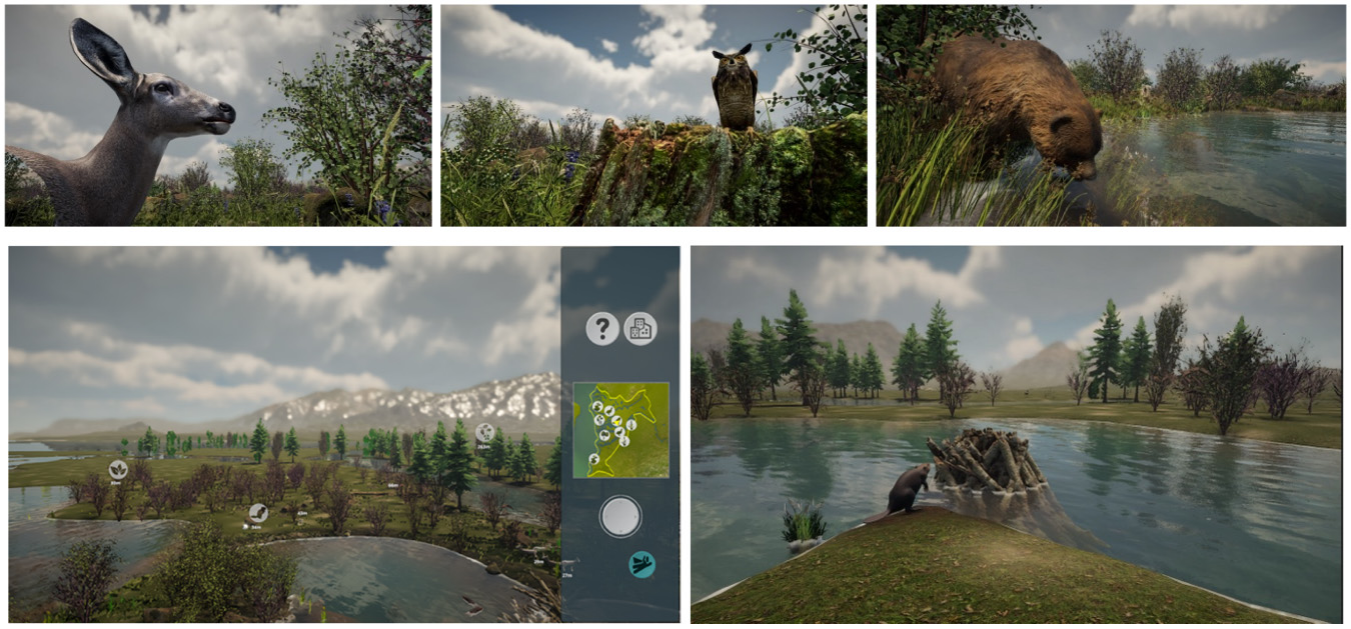
Waterways 3D visualization images of the Mill Creek ecosystem.

The Waterways interactive virtual 3D world of the pre-colonial Okanagan landscape was constructed using an amalgam of scientific, Indigenous, artistic, and human-centered techniques and perspectives (Figure 4). Historical visualizations were constructed based on Syilx Knowledge Keeper recollections, naturalist and historic agricultural records, Okanagan Historical Society documentation of land tenure, reconstructed terrestrial ecosystem mapping for 1800 and 1938, and geospatial, land use, and zoning data. With mapping data and a high-definition rendering pipeline, the research team generated a detailed map of the local topography and vegetation pre-contact and then used the Unity Game Engine with the GAIA Toolit to procedurally generate the terrain and populate plant species within defined microclimates. A large number of resources were compiled for accurate plant and animal models to be inserted into the 3D world. The team referenced a list of plants and animals from the Syilx En’owkin Centre and environmental history literature of the Okanagan, acquired available 3D resources to cover as much of the list as possible, and modified and built animal and plant assets for location-specific variations of Okanagan species.

### Collaborative research design and implementation

Strengthening cross-cultural and transdisciplinary understandings, collaboration, knowledge sharing, and multidirectional channels of communication on water responsibility was an explicit goal of Waterways. Collaboration between the researchers and the Syilx community was a prerequisite for cross-cultural understanding, and in turn, fostering understanding was a precondition of collaboration. Iterative shared working processes were central to Waterways, from conceptualization and design to museum production. For the first few years of the project, the research team spent most of its time within the Syilx community, shadowing practitioners at the ECOmmunity Place and participating in community activities such as traditional ceremonies. “Ask, ask, ask [the Syilx community],” “follow the [Syilx] people,” “shadow, record and learn [from Syilx community members]” became the research team's mantras over the 4-year project. Team members continuously asked: “what should we focus on?” “what do the [Syilx] people want?” and “how do we represent the [Syilx] people?” This immersion in the Syilx community pre-empted the conceptualization and design of Waterways.

Team members also participated in an enowkinwix^w^ community engagement process for an associated UBCO project, during which Syilx leaders articulated their research interests and needs.^1^ Enowkinwix^w^ is a traditional governance method used within the Syilx community that supports collaborative creativity, directs individualized positions toward a collective perspective, and builds solidarity for the benefit of all who are impacted or affected by the issue under discussion (Armstrong, 2009). Themes prioritized during the enowkinwix^w^ event with UBCO included exploring TEK (Syilx-led and controlled) and the relationship between Western science and TEK, building the capacity of Indigenous youth and scholars, and studying riparian zones, cottonwoods, salmon population restoration, grizzly bear habitats, and huckleberry. Waterways ultimately espoused several of these lines of inquiry consistent with Indigenous methodological, co-design, and justice design principles of focusing on the concerns of those most impacted by the outcome of the design and responding to the articulated interests of the Syilx community.

The composition of the Waterways research team, which, as noted above, comprised a large group of artists and scientists from UBCO, both Syilx and non-Indigenous, as well as community partners, reflected the aim of collaboration and transdisciplinary research. Waterways partners included the Syilx En’owkin Center, the Okanagan Nation Alliance, the Kelowna Museums Society, the Okanagan Collaborative Conservation Program, and the Okanagan Basin Water Board. At UBCO, Waterways worked out of the Centre for Culture and Technology to bring together researchers on complex environmental systems and adaptation, de-colonization and Indigeneity, biodiversity, and resilience.

An iterative co-creative process was applied throughout the research and design to capture the richness of cross-disciplinary practice. New knowledge and artwork developed for the museum exhibition, for example, evolved through collaboration between cross-functional team members. These teams convened regularly, usually weekly but often multiple times a week (conducted in person or via Zoom during the COVID-19 pandemic) and were guided by Waterways research and community partners. The partners met bi-monthly on average to assess progress, validate research findings and outputs, and offer feedback. Team meetings took place at various locations, depending on the nature of the work, including at the Syilx ECOmmunity Place or at restoration and riparian sites. Waterways served as a design container for transdisciplinary perspectives and sources of scientific, anthropological, artistic, and community knowledge, from which meaning and multiple ways of knowing were derived. The co-creation process deliberately emphasized the perspectives and knowledge of Syilx participants, acknowledging their profound connection to water and their extensive water-related knowledge, while also addressing historical imbalances of power. In alignment with Indigenous protocols, the Syilx community played a crucial role in validating the research findings and the materials prepared for the exhibition. Given that Syilx team members are employed by the En’owkin Center or the Okanagan Nation Alliance, and are integral members of Syilx bands, there was a constant and meaningful engagement with the wider Syilx community throughout the entire research process.

The museum exhibition was evaluated using a participatory visual matrix method, an approach that captures the shared experiences of participants and the collective impacts stimulated by the sensory material of the exhibition. The evaluation consisted of two events held at the Okanagan Heritage Museum exhibition hall, each involving 10–12 participants from the Syilx and non-Indigenous communities. The evaluation's participatory setting and collective reflection process resulted in the symbolization of imaginative and emotional reactions, which otherwise would not have been articulated consciously.

### An Indigenous paradigmatic design

Indigenous research methodologies are about understanding the paradigm, which means that the research flows from an Indigenous belief system and is inherently relational. The Waterways theoretical framework embodies a Syilx Indigenous belief system in its research protocols and exhibition. The creative artistic process, for example, was harmonized with Indigenous methodologies to form a holistic research framework and convey realities as relational and interconnected. The inquiry itself embraced Syilx values of relational accountability, responsibility, relevance, and reciprocal engagement with all living beings.

**Figure 5.**
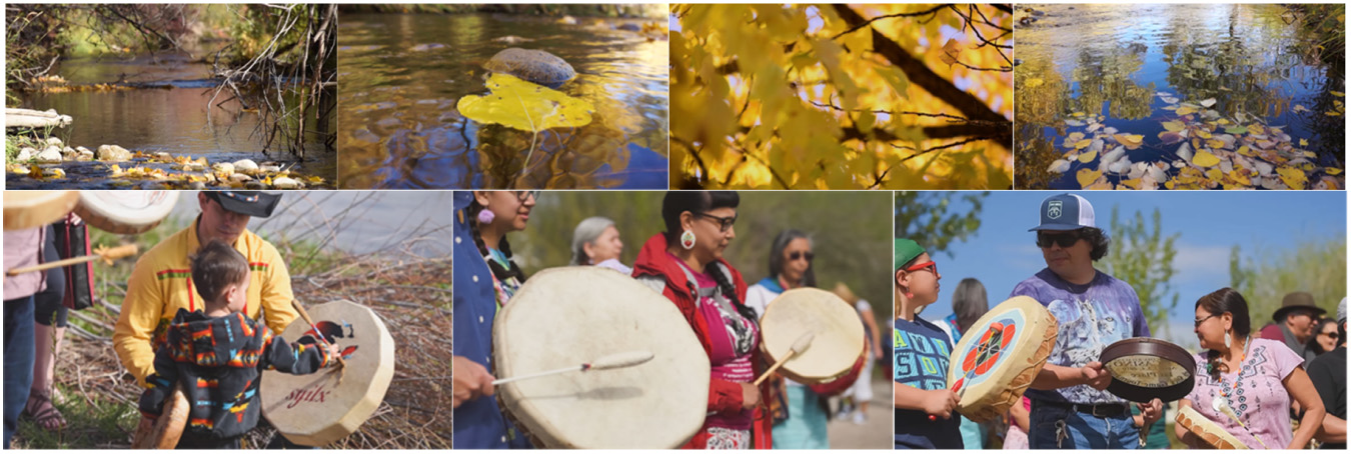
Photographs of cottonwood species and Syilx salmon ceremonies.

Indigenous methodologies similarly underscore research that respects Indigenous values. Data gathered for Waterways and subsequently used for the museum installation reflected the cultural protocols, values, and beliefs of the Syilx people. These included the needs of the whole living system of tmixʷ (which refers to the land and all life forms and relationships of a place); the belief that humans are an equal part of tmix^w^ along with all other living and non-living things; the non-negotiable obligation of Syilx stewardship of the tmix^w^, and the role of captikʷɬ (stories) as an ethical roadmap and system of intergenerational knowledge transmission (Armstrong, 2009,  2018). Indigenous values equating water with life and water as the most important element of all things in nature were reflected in the didactics on the exhibition's panels. The Syilx cultural significance of re-establishing the sockeye salmon population, the cultural imperative of restoring habitats, riparian systems, and biodiversity in the Okanagan, and the role of ceremony in strengthening community values, were also highlighted in the exhibition's audio and visual materials (Figure 5). Furthermore, Waterways artwork was conceptualized to create an imaginative space of engagement and reflection on multiple realities embedded in the “place” of the Okanagan.

Benefits to Indigenous communities are a central tenet of Indigenous methodologies. One of the significant contributions of Waterways was documented Indigenous plants and knowledge, including from elders interviewed by the project who have since passed on. This invaluable knowledge has now been meticulously preserved and archived at the Syilx En’owkin Center. Additionally, the project successfully developed 3D models of these plants in nsyilxcən (the Syilx language) and created detailed 3D visualizations of the Okanagan as it was before colonization. These detailed representations pay tribute to the ecological richness of the Okanagan and celebrate a place of profound importance to the Syilx people due to their deep and enduring connection to the land. Furthermore, members of the Syilx team highlight the value of Waterways as a powerful educational resource for Indigenous youth and children.

### Drawing on Indigenous knowledge(s) and Western science

Exploring the contemporary significance of Syilx TEK and how TEK and Western science complement one another was a Waterways research theme, following the request of Syilx community members during the enowkinwix^w^ event. These topics were subsequently probed in interviews, particularly in discussions related to the sockeye salmon return and the Syilx ECOmmunity Place's reestablishment of the Yellow-Breasted Chat in the Okanagan. The research revealed that bridging Indigenous and Western knowledge systems is increasingly valued. Interviewees stressed that applying TEK and Western knowledge can significantly relieve disruptions to the land, restore traditional socio-ecological relationships, and develop successful approaches to resilience in the face of climate change. Waterways also explored multiple “ways of knowing.” Interviews revealed that, for example, knowledge can come from a myriad of sources, including the oral tradition of intergenerational knowledge sharing, traditional teachings, empirical observation, and revelations. As explained by one Syilx Knowledge Keeper:

When I have a question, I ask myself, what do the Elders say? What do the stories tell us? What does the land or water say? It is a physical thing. When I’m out in my environment, I am conscious about the insects, bugs, dust, dirt and what they are telling me. It is also a metaphysical thing. What are my dreams telling me?

### A decolonizing and design-justice approach

A decolonizing perspective was intrinsic to Waterways. From its inception, Waterways challenged Western epistemological convention by bringing Indigenous methodologies to the inquiry, applying the enowkinwix^w^ process to identify research themes, having Syilx scholars lead different parts of the research, conducting interviews based on Syilx protocols, acknowledging the value and contributions of Syilx knowledge systems and bringing Syilx voices and “ways of knowing” to the museum audio and video installations and didactics. Conversations and exchanges conducted with the Syilx community, which were ultimately reflected in the museum exhibition, also created a space for discussing Syilx self-determination and transformation of practices and values vis-à-vis water. Furthermore, Waterways shed light on important issues related to Syilx governance in the analysis of sockeye salmon return to the Okanagan waterways. Research revealed, for example, that recognizing the Okanagan Nation Alliance as a legitimate government within the tripartite provincial-federal-Indigenous co-management framework, with equal decision-making powers among the three partners, was central to the success of sockeye salmon restoration.

Design justice principles were similarly present in the Waterways inquiry and museum exhibit. In addition to bringing multiple ways of knowing and skills to the design process, the inquiry brought awareness to the structural inequalities and mistreatment historically faced by the Syilx peoples. Interviewees spoke of the personal and collective impact of the Columbia Basin Water Treaty between Canada and the USA through stories of change and destruction of traditional water and land habitats. How the Syilx community endured and experienced injustice and racism through their restoration efforts and the resilience of the Syilx Nation in the face of oppression emerged during interviews and were reflected in the exhibition installation. One Syilx participant used the reestablishment of sockeye salmon as a metaphor for the return of the Syilx people:

The success of the chiefs following their vision, following their calling [to return the salmon]and following what was presented to them is a result of long and hard sacrifices with our ceremonies, of us saying we have resisted, despite legislation trying to wipe us out. The resistance: the resistance of the people in terms of secretly singing our songs, secretly speaking our language. But that's also in our captikʷɬ [stories] I know that's a natural part of us returning and coming back.

## Discussion

The Waterways 4-year research and museum exhibition on human-water relations in the Syilx Okanagan territory provides a real-life example of effective cross-cultural research that applied a mixed Western co-design and Indigenous methodological approach. Collaborative design principles reflected in Waterways included prioritizing design justice and incorporating reflexivity, flexibility, iteration, and emergence in the design process. From an Indigenous methodologies perspective, Waterways took an Indigenous “paradigmatic perspective,” in that the inquiry and exhibition flowed from the Syilx belief system with its worldviews and cultural values; acknowledged unique Syilx knowledge systems, which are oral, experiential, and holistic and transmitted through story; encompassed Indigenous “ways of knowing” and lived daily experiences; adopted a decolonizing framing to the methodology; and responded to Syilx needs and interests. Common to both methodological orientations is “relational practice,” which in Indigenous research is referred to as “self-in-relation” and “researcher in relation” and acknowledges the necessary subjective role of the researcher in the inquiry. Co-design research is inherently relational. As noted earlier, it is rooted in the practice concept of reflection-in-action and Schön's “reflective practitioner” in simultaneously integrating reflection with action and implementation.

An important methodological lesson arising from the Waterways mixed methodological approach was the need for the team to conform to culturally appropriate methods. This included framing the project based on Syilx-led ethical protocols of engagement and drawing from a traditional Syilx enowkinwix^w^ process to identify relevant research lines of inquiry, having the team participate in cultural ceremonies that hold meaning and significance to Syilx research partners, and applying methods that focused on Syilx oral accounts and stories. The immersive artistic exhibition design reflected these Syilx culturally appropriate methods with its inclusion of Indigenous voices, different ways of knowing, audiovisual communication elements, and a circular reflective inner space.

Another lesson from applying co-design and Indigenous methodologies in cross-cultural research is its time-intensive nature, which diverges from research funding cycles that tend to be short-term. This is particularly problematic when addressing inherently complex long-term issues related to climate and social change. In the case of Waterways, establishing relationships and building mutual understanding and trust with non-academic Indigenous research partners and fulfilling the obligations of Indigenous methodologies and co-design was time-consuming and risky. Continuously shadowing Indigenous Knowledge Keepers and experts on the land to learn about Syilx values, protocols, and practices and learn about Indigenous knowledge was central to the Waterways approach, but it also took time. Moreover, the research team had planned to co-design and build 3D visualizations of four Okanagan waterways; however, time pressures meant only one could be completed. Finally, as per Indigenous methodological principles, research outputs belong to the community where the inquiry is being conducted. For ethical reasons and to ensure accuracy and authenticity, the Waterways findings and exhibition content had to be validated and authorized by the community, which was also more meticulous and time-consuming than expected.

Finally, by applying a mixed Western co-design and Indigenous approach, Waterways was able to create an ethical space of engagement between the Syilx and non-Syilx communities. In the Indigenous context, ethical space is formed when “two societies, with disparate worldviews, are poised to engage each other” (Ermine, 2007: 193) and “where world views meet as equals, in which colonial hierarchies of knowledge systems (epistemologies) and world views (ontologies) are countered” (Nelson and Wilson, 2018: 21). Waterways, through its 4-year engagement and co-design process, was able to create this physical, psychological, and social ethical space through its introspection and reflection, dialogic engagement, relationship building, sharing of worldviews and ethics, learning and adaptation, and orientation toward justice and decolonization. Given the limited literature that exists on operationalizing ethical space concepts in Indigenous contexts (Nikolakis and Hotte, 2022), Waterways provides some insights on moving from concept to praxis.

## Conclusion

This article explores the application of co-design and Indigenous methodologies within “Waterways, Past, Present and Future”—an immersive media exhibition and research inquiry examining human-water relations in the Syilx Okanagan First Nation territory, BC, Canada. Amidst the nation's bumpy path toward reconciliation and the paucity of real-world experiences, Waterways emerges as a valuable model for cross-cultural collaboration in both research and co-design. By employing a mixed Indigenous-Western methodological framework, the project facilitated mutual understanding and helped bridge the gap in worldviews, values, and knowledge systems. The resulting exhibition's synergistic content, form, structure, audio-visual elements, and ambiance manifested a creative and technical amalgam of Indigenous and Western influences, underscored by shared values. Waterway's effectiveness can be attributed to the formation of a transdisciplinary team comprising both Syilx and non-Indigenous scholars, the application of a decolonizing approach to research, and the adoption of inquiry lines that bridged Syilx traditional knowledge with Western academic paradigms. Furthermore, a significant investment of time was made for place-based learning within the Syilx territory, fostering trust and cultivating relationships within the community.

Waterways, and initiatives of its kind, underscore the capacity of Indigenous communities to lead or co-lead research and co-design projects as well as the corollary challenges. Parallel examples from cross-cultural research partnerships in Indigenous settings, such as those involving the Rakiura Māori and Ngāti Hine tribes in New Zealand and the OurRiver Cooks River Sustainability Initiative, further enrich this narrative (Bos and Brown, 2012; PO’B, 2005). Together, these endeavors exemplify the potential of culturally responsive, Indigenous-led research to engender change, bridge diverse epistemologies, and cultivate understanding, and in doing so, contribute to the goal of reconciliation with Indigenous peoples.

## Notes

1 The full project name is Enhancing Ecosystem Sustainability: Contributing to reconciliation through the co-production of ecological knowledge from both Western and Syilx perspectives.
